# Recurrent giant retroperitoneal liposarcoma: Review of literature and a rare case report

**DOI:** 10.1016/j.amsu.2021.102329

**Published:** 2021-04-17

**Authors:** Lalith Kanthala, Samrat Ray, Sri Aurobindo Prasad Das, S. Nundy, N. Mehta

**Affiliations:** Department of Surgical Gastroenterology and Liver Transplantation, Sir Ganga Ram Hospital, New Delhi, 110060, India

**Keywords:** Retroperitoneal liposarcoma, De-differentiated, Recurrent, Surgical excision, Case report

## Abstract

**Introduction and importance:**

Retroperitoneal liposarcomas (RPLS) are usually grow large with frequent recurrences. Complete surgical excision remains the gold standard treatment for primary and even recurrent tumours. Their prognosis depends on their histological type and grade. We report a recurrent giant de-differentiated RPLS weighing 18.55 kg which was completely excised. To the best of our knowledge, this is one of the largest liposarcoma reported in the literature.

**Case presentation:**

A 40 year old female presented with a gradually progressing large abdominal lump for 1year. She had had a similar large lump twice in the past and undergone excision of the tumour elsewhere. Firm non-tender mass felt all over abdomen with edema noted over abdominal wall and bilateral lower limbs. PET CT showed large heterogeneously enhancing mass occupying almost the entire abdominopelvic cavity. 50 × 40 × 40cm tumour was completely excised and biopsy showed grade 2 dedifferentiated liposarcoma (DDLS). She is under close follow up with no recurrence at 12months.

**Clinical discussion:**

DDLS have lower risk of distant metastases but have a high risk of local recurrence. The most important favourable prognostic factor in these tumours is complete resection with negative margins. Because of the ineffectiveness of current chemotherapy and the requirement of intolerably high radiation doses, surgical excision remains the most effective treatment even for the localized recurrences of RPLS.

**Conclusion:**

The dedifferentiated subtype should be suspected in locally aggressive RPLS. Close follow up with early detection of recurrences and prompt excision with negative margins lowers the risk of recurrences and improves survival.

## Highlights

• Retroperitoneal liposarcomas are rare malignant tumours and are the most common sarcomas arising in this region.
• Prognosis depends on their histological type and grade.
• Surgical excision remains the most effective treatment even for localized recurrences.
• Chemo/radio therapy have very limited role in their treatment.

## 1 Introduction

This work has been reported as in line with the SCARE 2020 criteria [1]. Retroperitoneal sarcomas account for 10–15% of all soft tissue sarcomas [2]. Among them liposarcomas are the most common (20–45%); others include leiomyosarcomas, malignant fibrous histiocytomas and undifferentiated pleomorphic sarcomas [2,3]. Retroperitoneal liposarcomas (RPLS) are usually asymptomatic until they grow large enough to produce compression symptoms. Complete surgical excision remains the gold standard treatment for primary and even recurrent tumours as chemotherapy and radiotherapy have a very limited role [4,5]. Histological type and grade, further dictate the prognosis of liposarcomas as dedifferentiated, round cell and pleomorphic types are known for higher rate of recurrence and associated with a poor prognosis [6]. We report the case of a 40 year old female with a recurrent giant retroperitoneal dedifferentiated liposarcoma weighing 18.55 kg which was completely excised without removing any other abdominal organ. To the best of our knowledge, this is one of the largest liposarcoma reported in the literature. We believe that this case report helps to know more about this rare entity and it will be good for the researchers to review the literature regarding dedifferentiated liposarcoma.

## 2 Case report

A 40 year old female, suffering from hypothyroidism for 6 years, presented to our outpatient department with a gradually progressing large abdominal lump for 1year associated with bilateral pedal edema and difficulty in breathing for 1 month. She had had a similar large lump in 2013 and 2017, and had twice undergone excision of the tumour elsewhere. Biopsy revealed a well differentiated liposarcoma. She received 6 cycles of chemotherapy after the second surgery and her last cycle was in March 2018. She had not had radiotherapy. She was well till November 2018 when she again noticed a lump in the lower abdomen which gradually increased in size occupying her entire abdomen. There were no symptoms suggestive of bowel or urinary bladder involvement and no history of jaundice, back pain or headache.

**Fig. 1:**
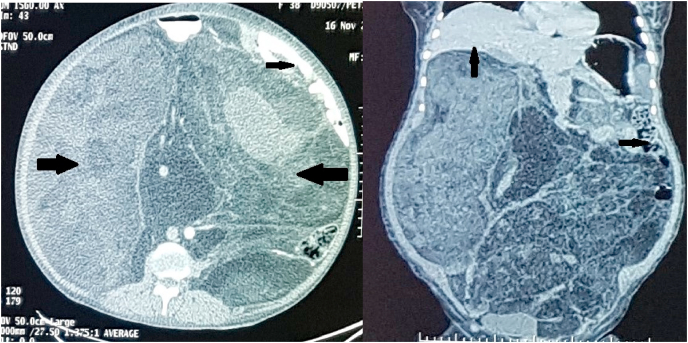
Cross-sectional and coronal CT images of the abdomen: large heterogeneously enhancing mass with a predominantly fat component occupying almost the entire abdominopelvic cavity (large arrows) with cranial displacement of the liver and small bowel in left hypochondrium (small arrows).

On examination, she was moderately built and nourished with weight of 83 kg, height of 161 cm and body mass index (BMI) of 32. She had a distended abdomen with a midline scar from a previous laparotomy. Firm non-tender mass felt all over abdomen with edema noted all over abdominal wall and bilateral lower limbs. Her bowel sounds were heard in the left upper quadrant of the abdomen. On digital rectal examination, a mass was felt anteriorly compressing the rectum. Her all lab parameters were within normal limits except for raised thyroid-stimulating hormone that was managed by increasing the dose of thyroxin from 25mcg to 50mcg once daily. She was evaluated with a PET CT (Fig. 1) which showed a mildly FDG avid, large heterogeneously enhancing lesion with a predominantly fat component occupying almost the entire abdominopelvic cavity with displacement of the other intra-abdominal organs. No other significant FDG avid lesion was seen elsewhere in the body.

**Fig. 2:**
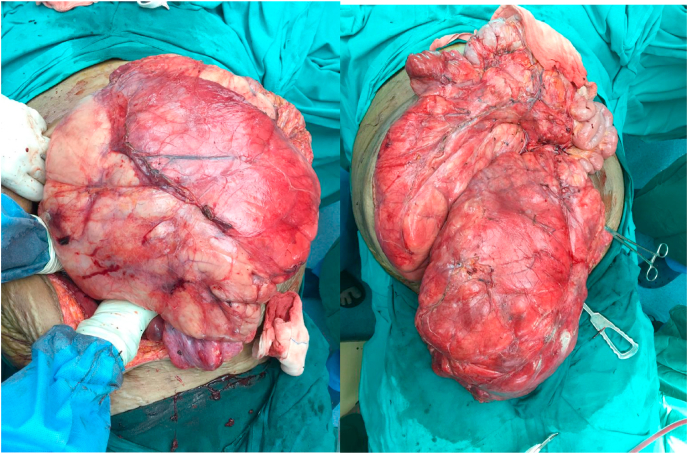
Intra operative images showing huge multilobulated encapsulated lipomatous tumor occupying the entire abdominal cavity.

As it was a second recurrence of the tumour and the patient refused neo-adjuvant therapy, it was decided to relieve her symptoms by surgical excision. On exploration by a senior consultant in surgical gastroenterology and liver transplantation department in this tertiary care center, a huge 50 × 40 × 40cm yellowish encapsulated multilobular retroperitoneal tumour mass was found extending from the diaphragm to the pelvis (Fig. 2). The entire small bowel and right-sided colon were pushed to the left hypochondrium with minimal interloop adhesions. The liver and right kidney were pushed cranially and medially. The tumour was completely excised without removing any other abdominal organ. The total weight of the excised tumor specimen was 18.55kg.

**Fig. 3:**
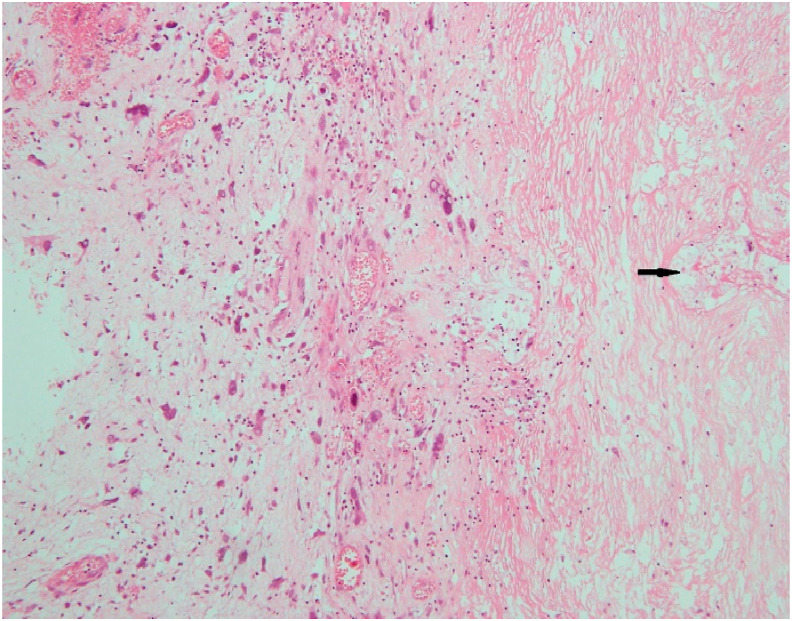
Photomicrograph showing a dedifferentiated area within the liposarcoma with pleomorphic tumor cells and mitotic activity along with areas of necrosis (arrow) (H&E stain, ×100 original magnification).

Her post-operative course was uneventful. At the time of discharge, 1 week after surgery, her weight was reduced to 61 kg with BMI of 23.5. The final histopathology report was suggestive of large grade 2, dedifferentiated liposarcoma (DDL) with undifferentiated pleomorphic sarcoma (UPS) features (Fig. 3), with less than 50% necrosis and a mitotic rate of 5–6/10hpf. pT4N0Mx (stage 3B according to AJCC staging system 8th edition). She is now under close follow up with no recurrence detected on a CT scan at 12months.

## 3 Discussion

Retroperitoneal liposarcomas (RPLS) are rare malignant tumours, representing between 0.07 and 0.2% of all neoplasms and are the most common sarcomas arising in this region [7]. The average age of presentation is between 40 and 60 years, with an equal sex distribution [8]. They are believed to be sporadic and thought to arise due to genetic abnormalities.

Most of the giant liposarcomas reported in the literature belongs to the dedifferentiated subtype [9,10] as the most common site for them being the retroperitoneum which has a large potential space, allows them to grow very big without being noticed and to become dedifferentiated over a longtime. Dedifferentiation occurs in up to 10% of liposarcomas [11].

A dedifferentiated liposarcoma (DDLS) is defined as a primary or recurrent well-differentiated liposarcoma (WDLS), with a region of abrupt transition to a non-lipogenic sarcoma [6]. Both fatty and nonfatty components are seen in the same tumour on CT or MRI. Macroscopically, they appear as large multinodular yellow masses with distinct tan-to-gray coloured non-lipomatous solid areas, which may contain areas of necrosis or haemorrhage.

DDLS have a lower risk of distant metastases than other high-grade liposarcomas but have a high risk of local recurrence. In a series of 65 primary retroperitoneal DDLS patients, the 3-year recurrence-free survivals observed were 17% for local and 70% for distal recurrences and the 5-year disease-specific survival was only 20% [12]. The most important favourable prognostic factor in these tumours is complete resection with negative margins. According to another series, R0 resection increases survival from 16.7% to 58% with a median survival of 103 months and 18 months in R1 and R2 resections respectively [7,8].

Mortality from RPLS is usually due to local recurrence as a result of incomplete resection. This occurs mostly due to difficulty in differentiating the tumour from adjacent normal fat and due to an inability to obtain a safe margin in the absence of a definite vascular-lymphatic peduncle of the tumour.[13] Recurrence of liposarcoma after initial surgery has been frequently observed within 6 months to 2 years with a rapid growth pattern with the mean tumour volume doubling time of about 100 days.[14].

Because of the ineffectiveness of current chemotherapy and the requirement of intolerably high radiation doses, surgical excision remains the most effective treatment even for the localized recurrences of RPLS. The possibility of complete surgical resection in patients with a first recurrence is up to 80%, however it decreases to 60–70% for the subsequent recurrences [6]. Surgery may be combined with neoadjuvant systemic therapy or intensity modulated radiotherapy depending on the histologic subtype, growth rate, and extent of disease.

All symptomatic local recurrences and asymptomatic recurrences which are impinging on critical structures or suspicious of dedifferentiation on CT scan with a growth rate of less than 1cm per month should be offered surgery. The asymptomatic local recurrences with a growth rate of more than 1 cm per month should be offered systemic chemotherapy or new targeted therapy trials [6].

## 4 Conclusion

Recurrence after excision of retroperitoneal liposarcomas is common. The dedifferentiated subtype should be suspected in cases which are locally aggressive. Close follow up with CT scans detects early recurrences and prompt reoperation with complete excision with negative margins lowers the risk of recurrences and improves survival.

## Conflicts of interest

None declared.

## Sources of funding

None.

## Ethical approval

Ethical approval was not required.

## Consent

Written informed consent was obtained from the patient for publication of this case report and accompanying images. A copy of the written consent is available for review by the Editor-in-Chief of this journal on request.

## Author contribution

Lalith Kanthala – Data Collection and analysis, Drafting article.

Samrat Ray, Sri Aurobindo Prasad Das – Study conceptualization and designing, Drafting.

Samiran Nundy, Naimish Mehta – Final Drafting.

## Research registration

N/a.

## Guarantor

Dr Lalith Kanthala.

## Provenance and peer review

Not commissioned, externally peer-reviewed.

## Declaration of competing interest

None.

## Registration of research studies

Name of the registry: N/a.

Unique Identifying number or registration ID: N/a.

Hyperlink to your specific registration (must be publicly accessible and will be checked):
